# Effects of BM-573 on Endothelial Dependent Relaxation and Increased Blood Pressure at Early Stages of Atherosclerosis

**DOI:** 10.1371/journal.pone.0152579

**Published:** 2016-03-28

**Authors:** Miguel Romero, Elvira Leon-Gomez, Irina Lobysheva, Géraldine Rath, Jean-Michel Dogné, Olivier Feron, Chantal Dessy

**Affiliations:** 1 Pole of Pharmacology and Therapeutics (FATH), Institute of Experimental & Clinical Research (IREC), Université Catholique de Louvain (UCL) Medical School, Brussels, Belgium; 2 Department of Pharmacy, University of Namur, Namur, Belgium; University of Southampton, UNITED KINGDOM

## Abstract

Endothelial dysfunction is considered to be an early event in atherosclerosis and plays a pivotal role in the development, progression and clinical complications of atherosclerosis. Previous studies have shown the beneficial effects of combined inhibition of thromboxane synthase and antagonism of thromboxane receptors by BM-573 on atherosclerosis; however our knowledge about the beneficial effects of BM-573 on endothelial function and increased blood pressure related to early stage of atherosclerosis is limited. In the present study, we investigated the effects of short-term (3μM, 1 hour) and chronic (10mg/L, 8 weeks) treatments with BM-573 on vasodilatory function, nitric oxide (NO) bioavailability, oxidative stress and systolic blood pressure in 15 weeks old apolipoprotein E-deficient (ApoE-KO) mice. ApoE-KO mice showed a reduced endothelium-derived relaxation. In addition, NO bioavailability was reduced and oxidative stress and blood pressure were increased in ApoE-KO mice versus wild-type mice. BM-573 treatments were able to improve the relaxation profile in ApoE-KO mice. Short-term effects of BM-573 were mainly mediated by an increased phosphorylation of both eNOS and Akt, whereas BM-573 *in vivo* treatment also reduced oxidative stress and restored NO bioavailability. In addition, chronic administration of BM-573 reduced systolic blood pressure in ApoE-KO mice. In conclusion, pharmacological modulation of TxA2 biosynthesis and biological activities by dual TP antagonism/TxAS inhibition with BM-573, already known to prevent plaque formation, has the potential to correct vasodilatory dysfunction at the early stages of atherosclerosis.

## Introduction

Numerous studies have emphasized the pivotal role of endothelial dysfunction in the development, progression or clinical complications of atherosclerosis [1, 2]. Endothelial dysfunction results from an unbalance between production and release of endothelial relaxing (NO, EDH(F), PGI_2_) and contracting factors (ET-1, TxA_2_ and PGs). Although the endothelium plays multiple functions, a reduced vasodilatory response to pharmacological stimulation constitutes a recognized indicator of endothelial dysfunction. Evidence of vasodilatory dysfunction has been clearly documented in plaque-prone vessels of human or in animal models of dyslipidemia [3], although it remains to be characterized in the resistance vasculature.

The formation of prostacyclin (PGI_2_), thromboxane (TxA_2_), and isoprostanes is markedly enhanced in patients with atherosclerosis [4, 5]. Activation of TxA_2_ receptors (TP receptors) causes potent vasoconstriction and induces increased formation of superoxide anions (O_2_^-^) and peroxynitrite (ONOO^-^), a product of rapid reaction of O_2_^-^ with nitric oxide (NO) that accelerates NO degradation and reduces its availability [6, 7]. In the last decades, numerous reports have suggested that TP receptor antagonism (with sulotroban or terutroban) or direct inhibition of thromboxane synthase (TxAS) (with furegrelate) can not only have antiplatelet effects but also impact endothelial dysfunction as well as the inflammatory component of atherosclerosis [8–11]. Failure to inhibit deleterious isoprostanes synthesis (known markers of oxidative stress and TP receptor agonists) explains why the latter drugs did not live up to the expectations in clinical trials. Therapeutic interest has thus switched to compounds that combine thromboxane synthase inhibition and TP receptor antagonism, such as BM-573. In previous *in vitro* and *ex vivo* studies, BM-573 has been demonstrated as a potent dual compound able to reduce TxA_2_ production by TxAS inhibition and to prevent the action of TxA_2_ by blocking the TP receptors [12, 13]. In addition to its antiplatelet and antithrombotic effects, BM-573 has proven to be effective in different animal models of cardiovascular diseases where levels of TxA_2_ are increased [14, 15].

The principal aim of this study was to evaluate the impact of dual TxAS inhibition/TP receptor antagonism by BM-573 on the endothelial dysfunction associated with hyperlipidemia by focusing on the eNOS/NO pathway. To decipher the beneficial properties of BM-573, we dissociated acute effects, associated to potential modulation of protein activity, from chronic effects, potentially linked to protein expression. Effects of BM-573 were measured on endothelial vasodilatory function, NO bioavailability, oxidative stress in resistance vessels from apolipoprotein E-deficient (ApoE-KO) mice at early stages of atherosclerosis. In this report, we demonstrate that both acute and chronic BM-573 treatments improve the endothelial dependent relaxation and prevent the increase of systolic blood pressure in ApoE-KO mice at early stages of atherosclerosis.

## Materials and Methods

### Animals and Experimental protocols

ApoE-KO male mice and their wild-type littermates (C57BL/6J or WT), were obtained from Charles River Laboratories (Belgium) and housed in a temperature controlled room with a 12:12 light-dark cycle and food and water ad libitum. After two weeks of acclimatization, experimental procedures were performed. For vascular reactivity studies and sample collection (aortae and blood), mice were euthanized by exsanguination under general anesthesia (Ketamine/Xylasine, 84 and 5mg/kg IP respectively). Telemetry catheters were implanted under general anesthesia (Ketamine/Xylasine, 84 and 5mg/kg IP respectively) and mice were given buprenorphine (0.1mg/kg SQ BID) during 5 days for analgesia.

To evaluate the acute effects of BM-573, mice (N = 8–12) were sacrificed at 15 weeks of age and their isolated vessels were incubated in absence or presence of BM-573 3μM for 1 hour in physiological salt solution buffer at 37°C. For chronic treatment, 7-weeks-old mice were randomized into two groups (N = 8–12 animals in each group) to receive either vehicle or BM-573 (10mg/L) *per os* for 8 weeks. The doses used were based on previously published studies [14, 15]. All experimental procedures and protocols were approved by the local Ethics Committee "Comité d'Ethique pour l'Expériementation animale", Secteur des Sciences de la Santé, Université Catholique de Louvain (agreement 2012/MD/UCL/004), according to National Care Regulations and Directive 2010/63/EU of the European Parliament and of the Council.

### Vascular reactivity studies

Mice were sacrificed and second branch mesenteric arteries were mounted on a pressure myograph as previously described [16]. Briefly, isolated micro-vessels (140–160μm) from Apo E-KO and WT mice treated or not with BM-573 were dissected under a stereoscopic microscope and mounted on a 110P pressure myograph (Ionoptix). Vessels were left to recover for 45–60 min in no-flow conditions (40mmHg, 37°C) in calcium-containing physiological salt solution buffer (PSS). Acute effects of BM-573 were measured in vessels isolated from control mice, exposed for 1h to BM-573 3μM (also named ex vivo or short-term treatment) while chronic effects were evaluated in vessels from mice treated for 8 weeks with BM573 10mg/L. In that case, the drug was excluded from all bathing solutions during the experimental procedure. Changes in the outer diameters were tracked and measured with the Myoview software.

### Nitric oxide availability assayed by electron paramagnetic resonance spectroscopy *in vivo* and *in situ*

At the end of the treatment, NO bioavailability was assayed by Electron Paramagnetic Resonance (EPR) spectroscopy in venous blood and isolated aortae from mice, as described previously [17]. Briefly, endothelial nitric oxide synthase (eNOS) activity was measured in isolated aortic rings by EPR spin trapping as concentration of paramagnetic NO adduct accumulated in tissue after stimulation by Ca(II) ionophore (ionomycin, 2μmol/L) at 37°C during 30 minutes in presence of spin trap (colloid form of [Fe(II)-(DETC)2], 0.5mmol/L in KREBS buffer), and was normalized by weight of dry aortae. The level of ferrous nitrosylated hemoglobin (Hb-NO) circulating in whole blood, was quantified from the EPR signal of 5-coordinate-α-Hb-NO using a hyperfine component of the spectrum (near g = 2.02) after subtraction of the EPR signal from protein-centered free radicals which overlapped with Hb-NO EPR signal. The EPR spectra were recorded by a Bruker EMX100 spectrometer (X-band, microwave frequency 9.35GHz, modulation frequency, 100kHz) with the following setting: microwave power (MP), 20mW; modulation amplitude (MA), 0.5mT; 10 scans, at 77K using finger dewar.

### Assessment of vascular ROS

Dihydroethidium (DHE), a sensitive superoxide anions (O_2_^-^) probe, was used to evaluate in situ production of vascular reactive oxygen species (ROS) in aortic tissues as described previously [18].

Quantitatively, basal SOD-sensitive ROS production was detected by EPR in isolated aortic rings using a spin probe *in situ*. Briefly, aortic rings were preincubated in KREBS-DTPA-Hepes buffer (0.1 mM DTPA, 20 mM HEPES, pH 7.5) in the presence or not of SOD (100 U/mL), inserted in capillaries (Blaubrand ®; 50 μl) immediately after addition of the ROS-sensitive spin probe (1-Hydroxy-3-methoxycarbonyl-2,2,5,5-tetramethylpyrrolidine, CM-H, Alexis Biochemical Inc.) as described previously [19]. Specific methodological details are provided in the S1 File. The kinetics of the CM· EPR signal formation was recorded on-line in capillary interposed into the cavity of the EPR spectrometer (MS400, Magnettech, X-band; microwave frequency 9.35GHz, modulation frequency, 100kHz) with following setting: MP, 20mW; MA, 0.1mT; 10 scans during 10 minutes at 37°C. The rate of the CM· radical formation was calculated using Magnettech kinetic software as a slope of linearized kinetic curve, obtained from automatical recording of the signal, and normalized by the length of aortic rings.

### Immunoblotting Experiments

At the end of the treatment, aortae were collected to evaluate Akt and eNOS phosphorylation by Western blotting, as previously described [16, 20]. Western blotting was also used to assess COX-2 protein expression in aorta homogenates. Specific methodological details are provided in the S1 File.

### Quantitative real-time PCR

Reverse transcription was done from total RNA using the SuperScript II RNase H− reverse transcriptase and random hexamer primers (Invitrogen). Quantitative real-time PCR (qPCR) analyses were done in triplicate using iQ SYBR Green Supermix (Bio-Rad). PCR fluorescence data were obtained and analyzed with the IQ5 instrument (Bio-Rad). Specific methodological details are provided in the S1 File.

### Analysis of blood pressure and heart rate by implanted telemetry

Systolic blood pressure (SBP) signal and heart rate (HR) were measured in conscious, unrestrained animals with surgically implanted, miniaturized telemetry devices (Datascience Corp., USA) as already described[17, 21]. Specific methodological details are provided in the S1 File.

### Statistical analysis

Relaxation to ACh were expressed as a percentage of the level of preconstriction induced by phenylephrine or KCl. For each concentration-response curve the maximum effect (Emax) was calculated using non-linear regression analysis (GraphPad Prism Software, San Diego, CA). All data are expressed as means ± SEM of experiments, with 'N' being the number of individual mice used in each experiment. Statistical analysis was done by comparing the curve obtained in resistance arteries treated with BM-573 with the control curve by means of a non-repeated measure analysis of variance (ANOVA) followed by Bonferroni post-hoc test. For NO production, oxidative stress, immunoblotting and qPCR experiments, the statistical analysis was done using one-way ANOVA followed by Newman-Keuls post hoc test. For assessing the effect of different treatments (BM-573 or vehicle) on blood pressure experiments, the statistical analysis was done using two-way ANOVA followed by Bonferroni post-hoc test.

## Results

### Endothelial dysfunction in ApoE-KO mice microarteries

**Fig 1:**
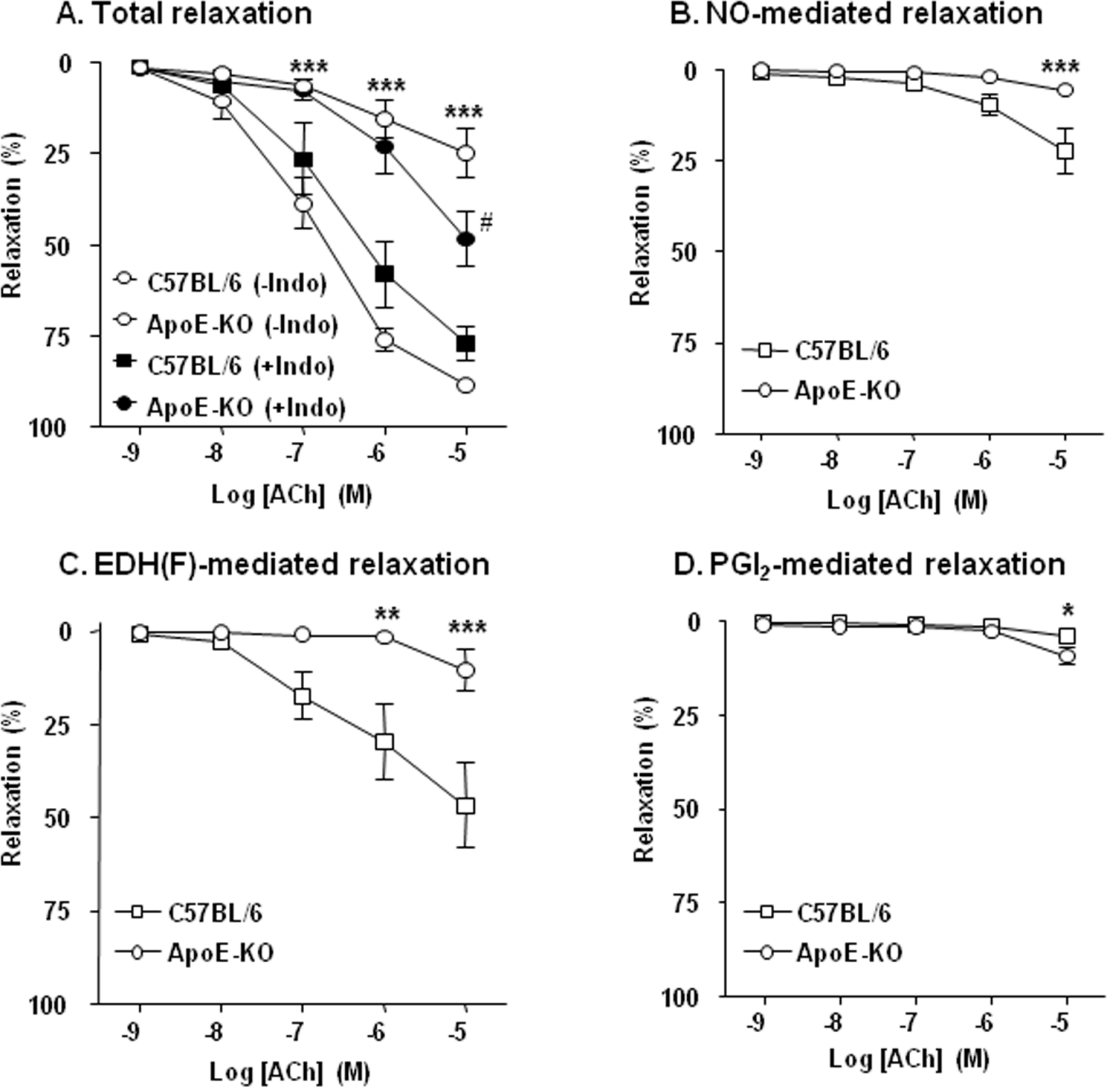
Characterization of endothelial dysfunction in ApoE-KO mice. (A) Acetylcholine (ACh) evoked relaxation in second branch of mesenteric arteries isolated from C57BL/6J and ApoE-KO mice after phenylephrine (Phe) contraction (10μM) in presence (filled) or absence (open) of indomethacin (10μM). Isolated relaxation pathways: (B) NO-mediated relaxation was evaluated in presence of indomethacin (10μM) after contraction with high-KCl solution (50mM). (C) EDH(F)-mediated relaxation was evaluated in presence of L-Ω-NoArg (100μM) and indomethacin (10μM) after contraction with phenylephrine (10μM). (D) PGI_2_-dependent relaxation was performed in presence of L-Ω-NoArg (100μM) after KCl (50mM) contraction. Results are expressed as mean ± SEM (N = 5–8 animals in each group). * P<0.05, ** P<0.01, *** P<0.001 ApoE-KO versus C57BL/6J mice, and # P<0.05 ApoE-KO (-Indo) versus ApoE-KO (+Indo)

A reduced acetylcholine (ACh)-evoked relaxation illustrates that resistance mesenteric arteries isolated from ApoE-KO mice showed an altered vasodilatory profile as compared to C57BL/6J mice (Fig 1A). To analyse this dysfunction and the effects of ApoE-KO genotype on the endothelium-dependent components of the relaxation, namely NO- and EDH(F)-mediated relaxation, experiments were performed in the presence or the absence of eNOS (L-Ω-NoArg) and cyclooxygenase (COX) (indomethacin) inhibitors as well as in conditions where endothelium-derived hyperpolarization was prevented (KCl 50mM). We showed that both the NO and EDH(F) portions of the relaxation to ACh were reduced in isolated vessels of ApoE-KO mice (Fig 1B and 1C). On the contrary, we observed that in response to ACh 10μM, the NO/EDH(F)-independent relaxation (assimilated to the COX-dependent relaxation) was significantly larger in mesenteric arteries from ApoE-KO mice than in wild type (WT) mice (18.3 ± 3.6% in ApoE-KO versus 9.0 ±4.2% in C57BL/6J; n = 4, P<0.05, Fig 1D). Additionally, preincubation with the COX inhibitor, indomethacin, significantly improved the acetylcholine-mediated maximal relaxation in ApoE-KO mice (Fig 1A), being without effect in isolated vessels from C57BL/6J mice. Altogether these results suggest that arachidonic acid-derived factors participate in the vasodilatory dysfunction observed in resistance arteries of ApoE-KO mice. Noteworthy, contraction levels were similar in all vessels independently of their origin (S1 Fig).

### Effects of BM-573 on ApoE-KO vasodilatory dysfunction

**Fig 2:**
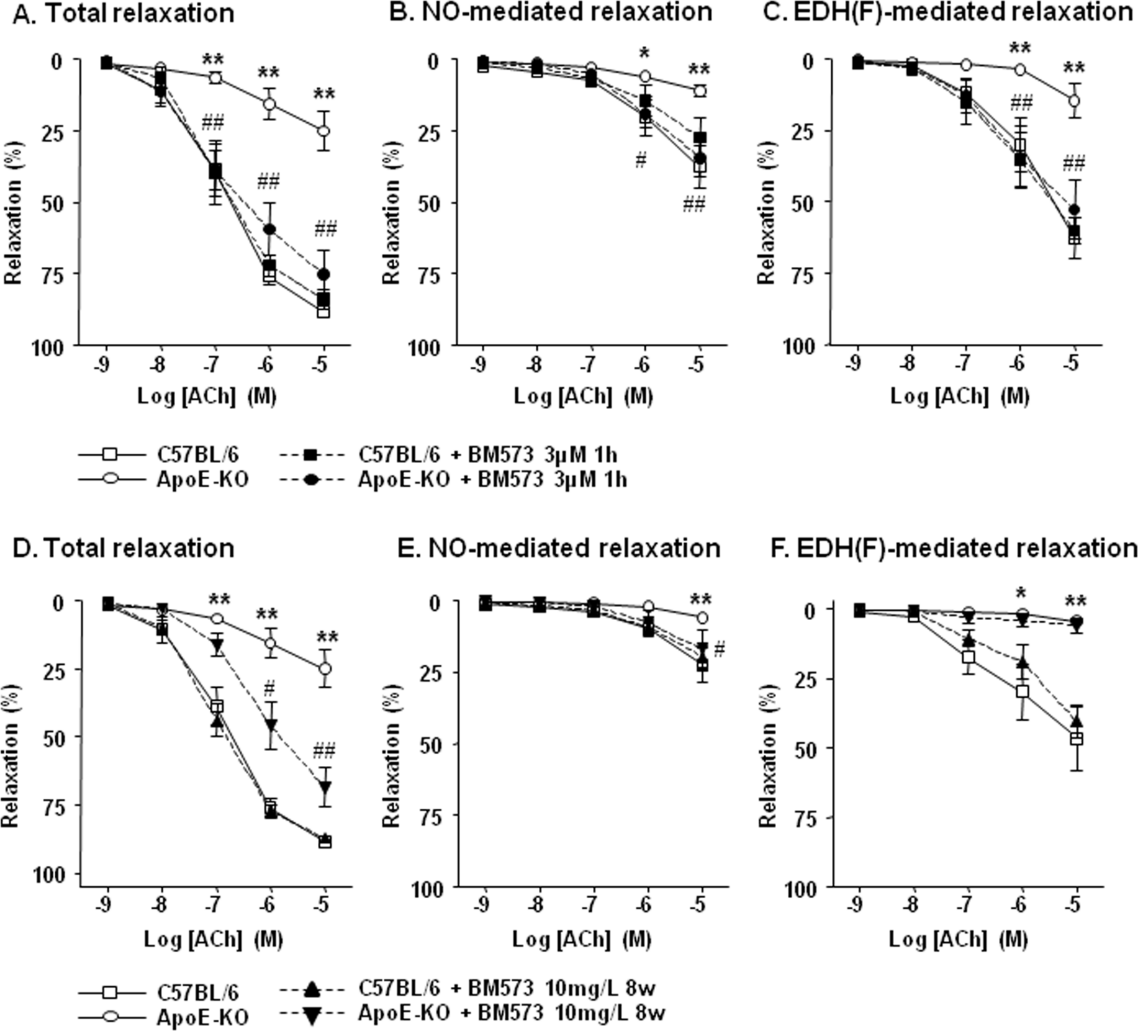
Effects of short *ex-vivo* and long *in-vivo* BM-573 treatment on the microvascular relaxation. (A, B and C) relaxation experiments were performed in microarteries isolated from 15-week-old mice preincubated in presence or absence of 3μM BM-573 during 1H (*ex-vivo*). (D, E and F) relaxation experiments were done in vessels isolated from 15-week-old mice that have been treated or not with BM-573 (10mg/L of drinking water) during 8 weeks. Acetylcholine-evoked relaxation was evaluated after phenylephrine or high-KCl solution (50mM) contraction to characterize: (A, D) Total-, (B, E) NO- or (C, F) EDH(F)-mediated relaxations in second branch of mesenteric arteries isolated from C57BL/6J and ApoE-KO mice treated or not with BM-573. Results are expressed as mean ± SEM (N = 5–8 animals in each group).* P<0.05, ** P<0.01 ApoE-KO versus C57BL/6J mice, and # P<0.05, ## P<0.01 ApoE-KO versus ApoE-KO+BM-573 treatment.

Effects of short-term treatment with BM-573 were evaluated in resistance mesenteric arteries exposed to BM-573 (3μM) for 1h versus controls. The BM-573- treatment improved the relaxation evoked by ACh in resistance arteries of ApoE-KO mice (Fig 2A) with a significant increase in both EDH(F) and NO components (Fig 2B and 2C). Whereas in microarteries of C57BL/6J mice, BM-573 acute treatment did not affect the ACh relaxing responses (see Fig 2A, 2B and 2C). As expected, in the presence of indomethacin, no effect of BM-573 could be measured on ACh relaxation in ApoE-KO mesenteric arteries (data not shown).

Effects of long-term treatment with BM-573 were measured in mesenteric microarteries isolated from mice treated with 10mg/L BM-573 *per os* from week 7 to week 15. Noteworthy, in order to characterize the chronic effects of BM-573 (in opposition to the acute responses measured in the short-term protocols), BM-573 was excluded from all bathing solutions during the experimental procedure. We observed that the total endothelium-dependent relaxation was increased in resistance arteries of ApoE-KO mice after *in vivo* treatment with BM-573 (Fig 2D). To specifically assess the effect of chronic BM-573 treatment on the NO component of the relaxation, vessels were incubated with indomethacin and preconstricted with a high KCl solution. In these conditions, a significant improvement of the ACh-evoked relaxation was observed in resistance mesenteric arteries collected from BM573-treated ApoE-KO mice (Fig 2E). In conditions where vessels were incubated with NOS and COX inhibitors and preconstricted with phenylephrine (Phe), the relaxation to ACh of microarteries isolated from BM-573-treated ApoE-KO mice remained significantly altered (vs control mice vessels) (Fig 2F), suggesting that the EDH(F) component of the relaxation is not involved in the beneficial effects of *in vivo* BM-573 treatment. Again, contraction levels were similar in all vessels independently of their origin or treatment (S1 Fig).

### Effects of BM-573 on NO production

**Fig 3:**
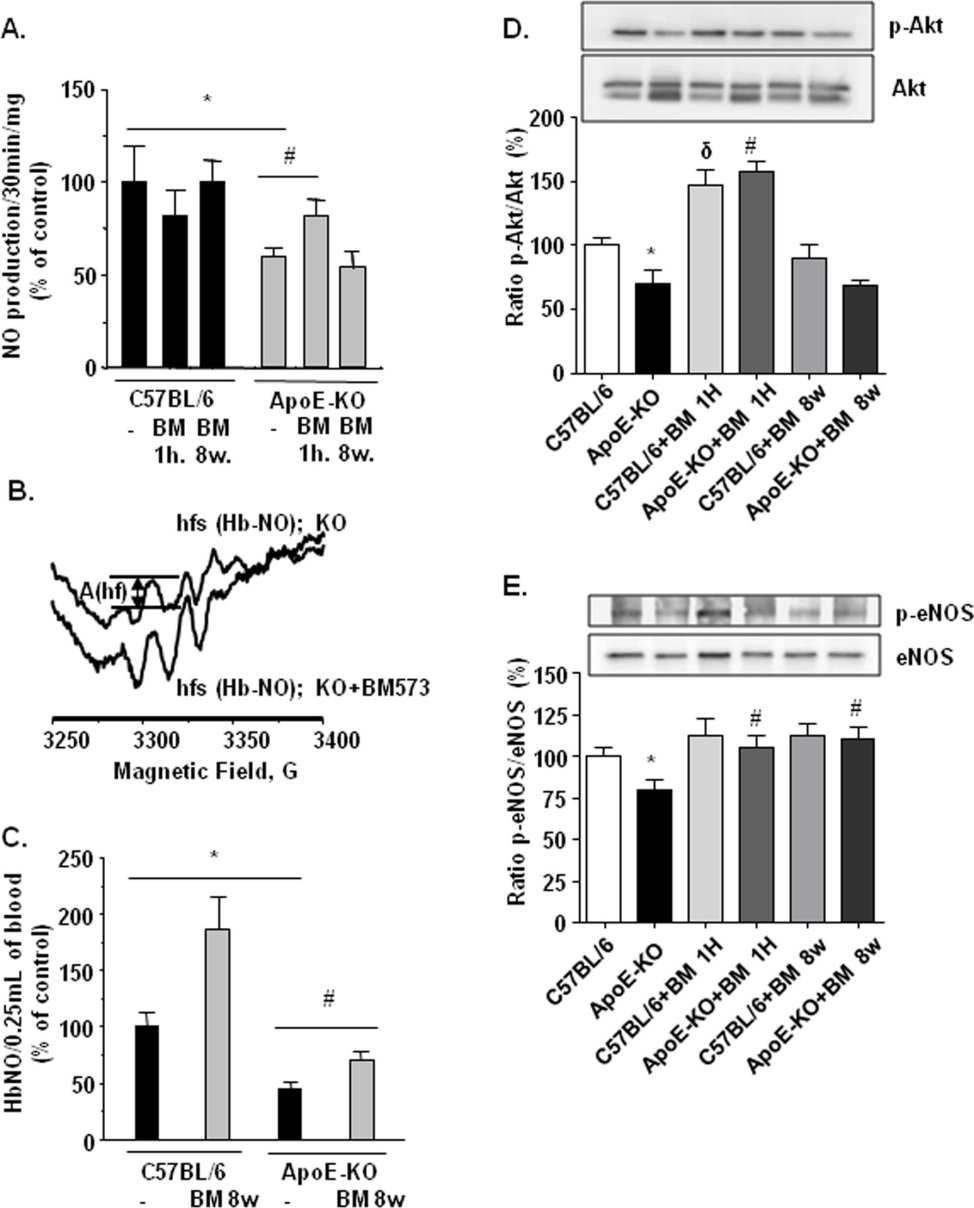
Effects of BM-573 on NO availability and eNOS-derived production pathway. (A) Ionomycin (2μM) stimulated NO production was measured by EPR spin trapping in isolated aortic rings from ApoE-KO and C57BL/6J mice treated or not with BM-573. The EPR signals of accumulated [Fe(II)NO-(DETC)_2_] complex were normalized to dry tissue weight (N = 4–6 animals in each group). (B, C) NO bioavailability was measured as level of Hb-NO complex assayed by EPR spectroscopy in venous blood: (B) typical EPR spectra of Hb-NO, presented as high-field component of the signal (hfs) after subtraction of protein-centered free radical signal; (C) quantitation of Hb-NO in whole blood of ApoE-KO mice treated or not with BM573 (N = 6–12 animals in each group). (D, E) Phosphorylation of Akt (Ser 473) and eNOS (Ser1177) measured by western blot in aorta homogenates. Results are expressed as mean ± SEM (N = 8 animals in each group). * P<0.05 ApoE-KO versus C57BL/6J mice, δ P<0.05 C57BL/6J versus C57BL/6J+BM-573 treatment and # P<0.05 ApoE-KO versus ApoE-KO+BM-573 treatment.

NO production was measured by EPR in isolated aortae from WT and ApoE-KO mice treated or not with BM-573. Ionomycin-stimulated NO production was significantly depressed in aortae coming from ApoE-KO mice (Fig 3A). While chronic BM-573 treatment did not influence the extent of stimulated NO production, short-term BM-573 treatment improved the NO-production in aortae of ApoE-KO mice, without influence on NO release in C57BL/6J aortae (Fig 3A). Circulating NO bioavailability, assessed by EPR as the level of nitrosylated hemoglobin (Hb-NO) in whole venous blood, was also reduced in ApoE-KO mice (Fig 3B and 3C). Interestingly, chronic treatment with BM-573 evoked a significant (P<0.05) increase in blood Hb-NO levels both in WT and ApoE-KO mice (Fig 3C).

In order to evaluate the effects of BM-573 on eNOS signaling pathway, we measured the levels of eNOS and Akt phosphorylation (Ser1177 and Ser473 respectively) in WT and ApoE-KO mice aortae. Both p-Akt/Akt and p-eNOS/eNOS ratios were significantly reduced in vessels from ApoE-KO mice versus WT mice (Fig 3D and 3E). Short-term treatment with BM-573 induced a significant increase in both eNOS and Akt phosphorylation in ApoE-KO vessels, while chronical *in vivo* treatment with BM-573 cause a small but significant (P<0.05) increase in eNOS phosphorylation in aortae of ApoE-KO mice (Fig 3D and 3E).

### Effects of BM-573 on oxidative stress

**Fig 4:**
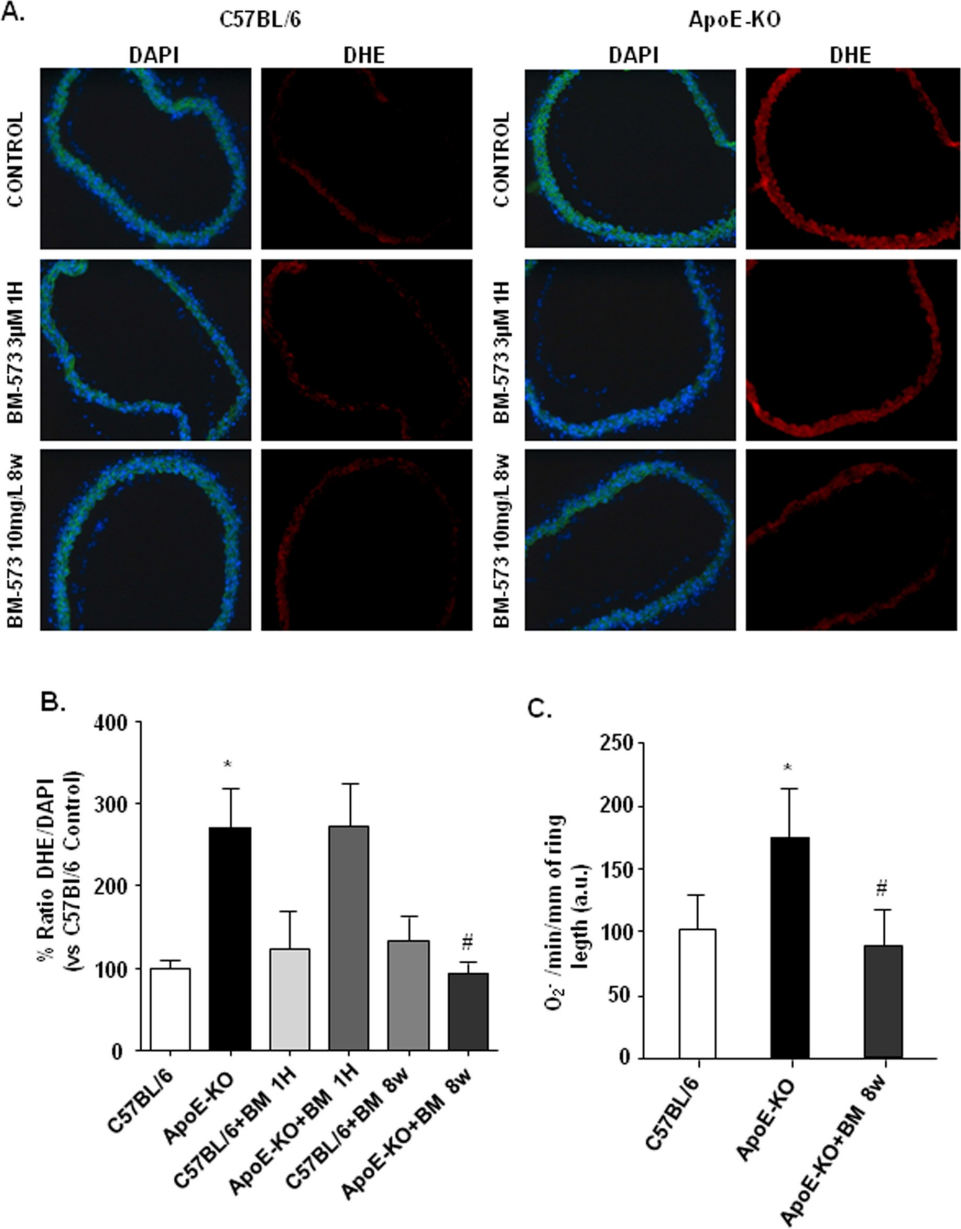
Effects of BM-573 on superoxide anions (O2-) production. (A) Dihydroethidium (DHE) (right) and nuclear DAPI (left) staining in mice aortae. (B) Values of red ethidium fluorescence normalized to blue DAPI fluorescence. (C) Basal O_2_^-^ production, assayed by EPR spectroscopy using CM-H spin probe. Results are expressed as mean ± S.E.M. of four different sections obtained from each aortic ring (N = 8–12 animals in each group). * P<0.05 ApoE-KO versus C57BL/6J mice and # P<0.05 ApoE-KO versus ApoE-KO+BM-573 treatment.

Oxidative stress was evaluated in aortic samples using dihydroethidium (DHE) staining (Fig 4). Aortic rings from ApoE-KO mice presented a significant increase in DHE/DAPI ratio, indicating an increased O_2_^-^ production (Fig 4A and 4B). While acute BM-573 treatment failed to modify the DHE-positive signal, long-term treatment reduced the increased DHE staining in aortic rings of ApoE-KO mice to a similar extent as that observed in aortae from WT mice (Fig 4A and 4B). These results were supported by direct measurements of O_2_^-^ using *in situ* EPR in isolated aortic rings from WT and Apo-E KO mice (Fig 4C). We also confirmed that *in vivo* BM-573 administration was able to prevent the increased O_2_^-^ production observed in ApoE-KO mice (Fig 4C).

**Fig 5:**
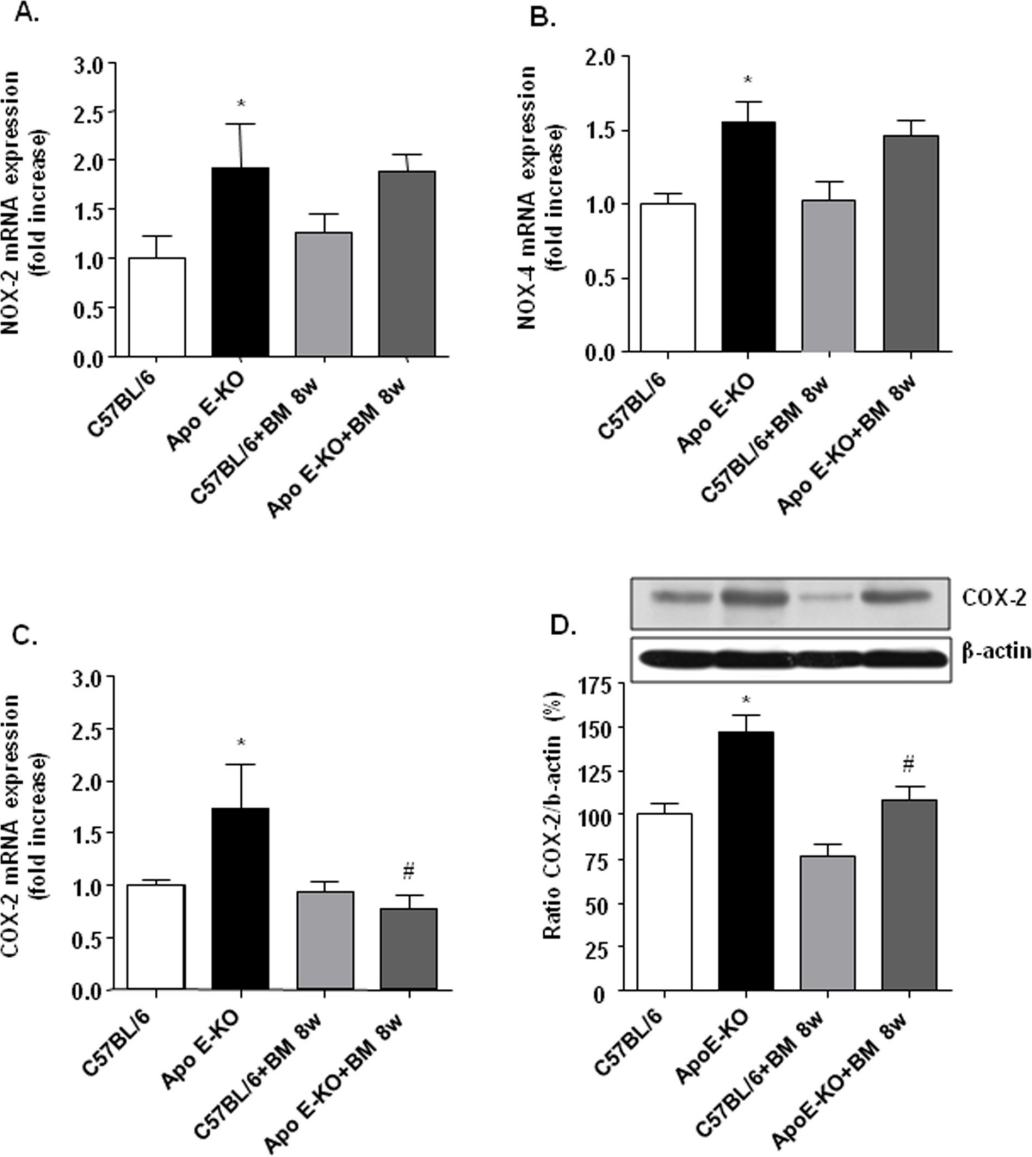
Effects of BM-573 on NOX-2, NOX-4 and COX-2 expression. Quantitative real-time PCR-based analysis of NOX-2 (A), NOX-4 (B) and COX-2 (C) expression in aortic homogenates from C57BL/6J and ApoE-KO mice treated or not with BM-573. (D) COX-2 protein expression assessed by western blotting. Results are expressed as mean ± SEM (N = 3–6 animals in each group). * P<0.05 ApoE-KO versus C57BL/6J mice and # P<0.05 ApoE-KO versus ApoE-KO+BM-573 treatment.

To evaluate the effects of BM-573 on the main sources of ROS in vascular tissues from ApoE-KO mice, namely uncoupled eNOS and NADPH oxidase [22–24], eNOS monomer/dimer ratio (as a marker of eNOS uncoupling) and NOX mRNA levels were quantitated in mice thoracic aortae. While we were not able to document eNOS monomer/dimer ratio from isolated vessels homogenates by Western blotting for technical reasons, NOX-2 and NOX-4 mRNA were increased in Apo-E KO mice compared to WT mice (Fig 5A and 5B), and NOX-1 mRNA levels were similar in tissues from all experimental groups (data not shown). BM-573 *in vivo* treatment however failed to influence either NOX-2 or NOX-4 up-regulation (Fig 5A and 5B).

### Effects of BM-573 on COX-1 and COX-2 isoforms expression

In resistance vessels from ApoE-KO mice, we observed an up-regulation of COX-dependent tone modulation (see Fig 1) as illustrated by an improved PGI_2_-associated relaxation. This was counter-balanced by an even more severe COX-dependent inhibition of NO/EDH(F) vasodilation. In agreement, the expressions of COX-2 mRNA and protein were higher in 15-weeks-old ApoE-KO mice than in WT mice. After 8 weeks of BM-573 *in vivo* treatment, vascular COX-2 mRNA expression was downregulated in ApoE-KO mice to a similar extent as that observed in WT mice (Fig 5C). These results were confirmed at the protein level by Western blotting (Fig 5D). In contrast, the expression of COX-1 mRNA was unmodified among the experimental groups (S2 Fig).

### Effects of BM-573 on increased blood pressure in ApoE-KO mice

**Fig 6:**
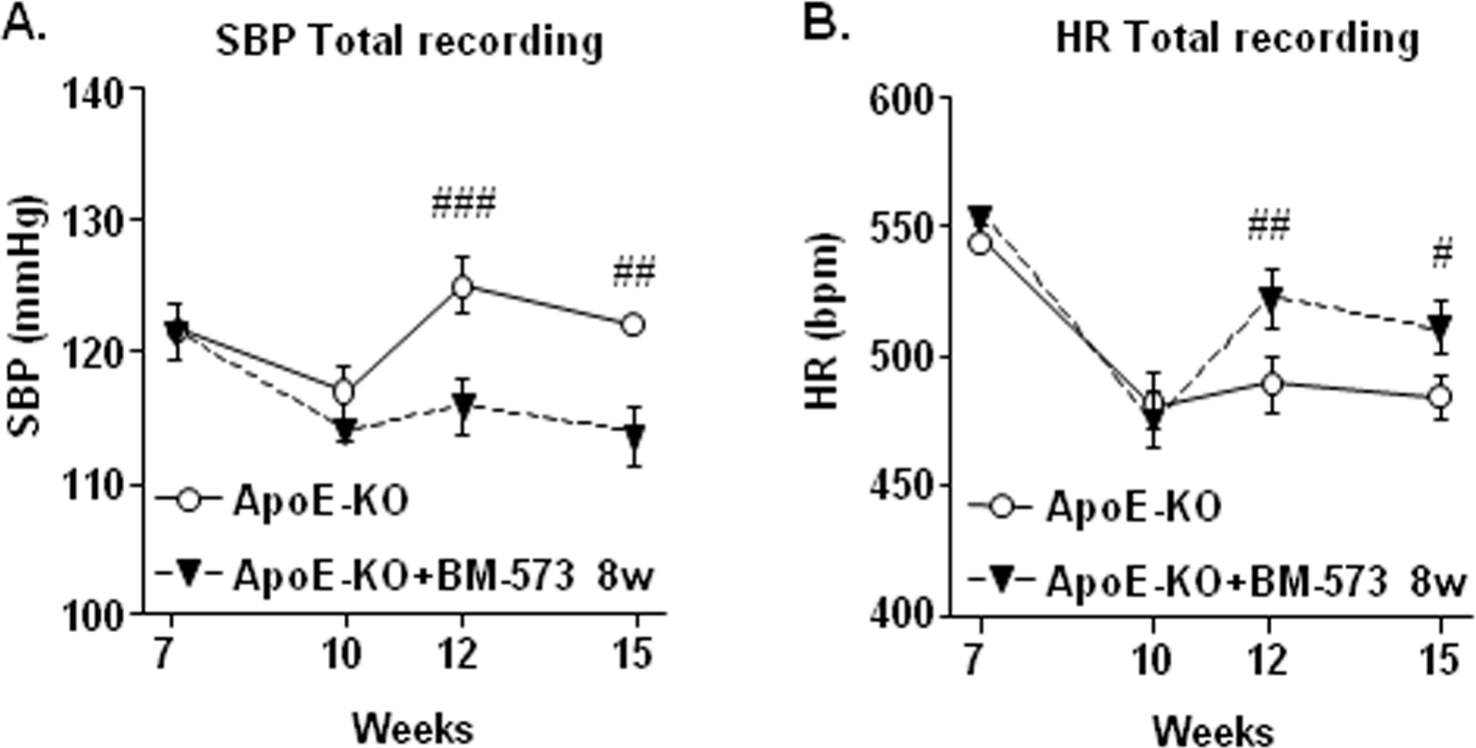
Effects of BM-573 on systolic blood pressure (SBP) and heart rate (HR). Effects of long-term BM-573 administration on systolic blood pressure (SBP) (A) and heart rate (HR) (B) measured by telemetry in ApoE-KO mice. Values are expressed as mean ± SEM (N = 4–6 animals in each group). # P<0.05, ## P<0.01 and ### P<0.001 ApoE-KO versus ApoE-KO+BM-573 treatment.

BM-573 treatment significantly reduced the increase in systolic blood pressure (SBP) and, conversely, increased the reduced heart rate (HR) observed in ApoE-KO mice (Fig 6).

## Discussion

The present study was designed to evaluate the beneficial effects of BM-573, a dual TxAS inhibitor and TP receptor antagonist, on the vasodilatory function and blood pressure in ApoE-KO mice at early stage of atherosclerosis. First, our results showed that resistance microarteries from ApoE-KO mice present an impairment of the vasodilatory response independently of the onset of plaque formation. This dysfunction arises from a defect in both NO- and EDH(F)-mediated vasodilation combined to an altered prostanoid balance. Second, we demonstrated that the inhibition of the TxA2 pathway by BM-573 prevent the altered relaxing response observed in the vasculature from ApoE-KO mice, through the restoration of NO bioavailability. In addition, *in vivo* telemetry documented the normalization of systolic blood pressure in BM-573-treated ApoE-KO mice. Although the underlying mechanisms seem to be different for short- and long-term BM-573 administration, a potentially additive mechanism could account for the beneficial effects of BM-573 in ApoE-KO mice at early stage of atherosclerosis.

ApoE-KO mice develop severe hypercholesterolemia and atherosclerotic lesions mimicking atherogenesis in humans. In addition, endothelial dysfunction and an elevated production of numerous arachidonic acid-derived mediators including TxA2 and prostaglandins have been described [25]. Although previous works have shown an impaired ACh response in ApoE-KO arteries, these studies were performed in older ApoE-KO mice (> 20 weeks) fed with high-fat diet to accelerate vascular atherosclerotic lesions formation. In these animals, endothelial dysfunction was evidenced only in plaque-prone vessels such as aorta or carotid artery [26, 27]. Interestingly, our results demonstrate an altered relaxation in response to ACh in resistance mesenteric vessels from young ApoE-KO mice under regular diet, where structural vascular changes and atherosclerotic lesions have not yet developed [28, 29]. This observation emphasizes the role of vasodilatory dysfunction as an early marker of atherosclerosis and a contributor to its pathogenesis. We found that the two major endothelium-dependent relaxing pathways, namely nitric oxide (NO) and endothelium-derived hyperpolarizing factor (EDH(F)), are altered in the vasculature of these young ApoE-KO mice. A reduced NO-dependent vasodilation is in line with early reports of endothelial dysfunction in resistance vessels from hypercholesterolemic patients [30]. The role of EDH(F) in pathologic vessels is more controversial considering that some reports mention a minimal alteration while others described an enhanced endothelium-dependent hyperpolarization and dilation in response to ACh. Whether the stage of atherosclerotic disease could account for these discrepancies requires further investigation.

Increased TxA_2_ production and TP receptor activation induce an impairment of endothelium-dependent relaxation and increase of reactive oxygen species [6, 7]. Previous studies have also shown that independently, TP receptor antagonists and TxAS inhibitors were able to improve endothelial function in different vascular diseases [31–33]. BM-573, a potent dual TxAS inhibitor and TP receptor antagonist [12], has shown to inhibit the development of vascular atherosclerotic lesions in different animal models of atherosclerosis [14, 15]. Specifically in ApoE-KO mice, BM-573, but not ASA, significantly decreased atherosclerotic lesions formation, showing that a dual TxAS inhibition and TP receptor antagonism is more effective in delaying atherosclerosis than single inhibition of TxA2 formation. Such benefit could be attributed to the antagonism of BM-573 on TP receptors, and the inhibition of deleterious effects from COX-independent such as isoprostanes. The current study now identifies the restoration of the eNOS/NO pathway as a major mechanism supporting the effects of dual inhibition of TxAS and TP receptor blockade in ApoE-KO mice at early stage of atherosclerosis. Whether this dual inhibition is superior to pure TP receptor antagonism is still unknown. Based on previous studies, we could speculate that blocking TxA2 synthesis might promote the accumulation of prostaglandin H2, and the synthesis of vasculoprotective prostanoids such as PGI2, due to BM-573 reduced TxA2 but fully preserved prostacyclin biosynthesis [12–14]. However, this was not evaluated in our study.

Among the underlying mechanisms of reduced NO availability in atherosclerosis, both the impaired enzymatic activity of eNOS and the accelerated degradation of NO by O_2_^-^ are proposed to play central roles [34–36]. Here, we showed that stimulated NO production and availability were significantly depressed and Akt-eNOS pathway altered in aortic rings from ApoE-KO mice. Furthermore, it has been described that TP receptor activation impairs endothelial NO-dependent vasorelaxation through the inhibition of both basal and stimulated Akt and eNOS phosphorylation via a Rho kinase-dependent mechanism [37, 38]. In our study, short exposure to BM-573 improved ACh relaxation and stimulated NO-production in ApoE-KO arteries together with an increased phosphorylation of both Akt and eNOS. Surprisingly, acute treatment with BM-573 also increased both Akt and eNOS phosphorylation in WT mice. This effect in WT mice could be due to blockade of TP receptor, which might protect basal phosphorylation of Akt in endothelial cells and in turn improve eNOS phosphorylation. Moreover, our data also indicate that in the long-term, BM-573 can restore NO availability in ApoE-KO mice through oxidative stress reduction. BM-573 chronic treatment reduced O_2_^-^ production in aortic rings from ApoE-KO mice and enhanced circulating NO levels in both WT and ApoE-KO mice, strongly suggesting that the observed increase in NO availability is associated with reduced NO degradation. Consistently, a rise in O_2_^-^ production has been demonstrated to accelerate NO degradation in both human and animal atherosclerotic vessels [39, 40]. Numerous oxidase systems might contribute to enhanced ROS levels in the vasculature, notably a PKC-dependent activation of NADPH oxidase. Here, we found a consistent up-regulation of NOX-2 and NOX-4 in aortae of ApoE-KO mice. However, BM-573 chronic treatment did not prevent NOX upregulation. Additionally, activation of COXs is also a source of O_2_^-^ due to the COX-dependent production of arachidonic acid-derived mediators, such as TxA2 and 8-isoprostanes, which activate TP receptors and may contribute to the development of endothelial dysfunction in both human and animal models of atherosclerosis [41–44]. In the present study, we found that COX-2 mRNA and protein expressions were increased in ApoE-KO mice and that BM-573 chronic treatment was able to prevent this rise. This is further supported by the greater COX-related modulation of tone observed in ApoE-KO mice (see Fig 1A and 1D). Although our results are in apparent contradiction with data obtained with another dual TP receptor antagonist and TxAS inhibitor on human umbilical vein endothelial cells [45], we cannot exclude that a modulation of COX2 expression in smooth muscle cells could explain our results as they were obtained from whole vessels homogenates. Moreover long-term treatment with BM-573 was also associated with a significantly enhanced p-eNOS/eNOS ratio. This, however, might reflect a different reality than that observed after short-term exposure to BM-573 and could account for a moderate phosphorylation of larger amounts of coupled eNOS. For technical reasons, we failed to document eNOS uncoupling as previously reported by others in a TxAS-dependent model [7]. Reduced oxidative stress by BM-573 and abrogation of the vicious circle that lead to eNOS uncoupling could however not be discarded, specifically for the BM-573 chronic treatment. An increased proportion of coupled eNOS, that is only moderately activated through phosphorylation would translate in an increased p-eNOS/eNOS ratio in denaturing conditions, an insignificant ionomycin-stimulated NO production in isolated vessels and would generate a mild but significant increase in circulating NO in chronically BM-573 treated mice.

Finally, we evaluated whether treatment with BM-573 could prevent the progression of hypertension observed in ApoE-KO mice. Although a controversy remains concerning the development of hypertension in ApoE-KO mice [21, 46, 47], we analyzed the effects of BM-573 on blood pressure in young ApoE-KO mice through direct arterial pressure measurements by telemetric recordings. In agreement with our previous results, ApoE-KO mice presented a significant and time-dependent increase of systolic blood pressure concomitant to a slight decrease of heart rate. Consistently with the improved vasodilatory profile afforded by both acute and chronic BM-573 treatments on the NO pathway, we showed that BM-573 was able to restore systolic blood pressure and heart rate of ApoE-KO mice. However, BM-573 had not effect on blood pressure variability (specifically the very low frequency VLF band (S3 Fig), indicating that BM-573 failed to restore the general alterations of neurohumoral control in ApoE-KO mice, as described previously [21]. These results indicate that prolonged treatment with a dual TxAS inhibitor/TP receptor antagonist represents a realistic approach to protect the vasculature against endothelial dysfunction and increased blood pressure, both well-established risk factors for the development and progression of atherosclerosis.

In conclusion, our study shows that BM-573 prevents and corrects the vasodilatory dysfunction in resistance arteries at early stage of atherosclerotic lesions by promoting eNOS activity and reducing oxidative stress. Together with a previous report showing plaque progression prevention by BM-573 in conductance vessels in the same mouse model [14], these data provide additional rationale to combine antagonism of TP receptors and TxAS inhibition as a therapeutic modality to prevent the vascular deleterious consequences of atherogenesis.

## Supporting Information

S1 Fig Contractile response of superior mesenteric arteries isolated from C57BL/6 and ApoE-KO. (A,D) Contractile response to KCl (50mM) or (B-F) phenylephrine (Phe) (10μM) in presence or absence of indomethacin (10μM) was measured in resistance mesenteric arteries from 15-week-old mice treated or not with BM-573 either per os during 8 weeks (10mg/L) or *ex-vivo* for 1H (3μM). No significant variations have been recorded in the contraction levels. Results are expressed as mean ± SEM (N = 5–8 animals in each group).
(TIF)

S2 Fig Effects of BM-573 on COX-1 expression. Quantitative real-time PCR-based analysis of COX-1 expression in aortic homogenates from C57BL/6J and ApoE-KO mice treated or not with BM-573. Results are expressed as mean ± SEM (N = 3–5 animals in each group).
(TIF)

S3 Fig Effects of BM-573 on blood pressure variability. Effects of long-term BM-573 administration on blood pressure variability, specifically the very low frequency VLF band, measured by telemetry in ApoE-KO mice. Values are expressed as mean ± SEM (N = 3–5 animals in each group).
(TIF)

S1 File Supplemental Materials and Methods. This complete supplemental information file contains: (i) Drugs and buffers information; and (ii) methodological details relating to measurements of superoxide anion production by EPR, western blotting, quantitative real-time PCR and blood pressure and heart rate monitoring.
(DOC)
