# Supplementary figures and images for: Effects of BM-573 on Endothelial Dependent Relaxation and Increased Blood Pressure at Early Stages of Atherosclerosis
Source: PLoS One. 2016 Mar 28;11(3):e0152579. doi: 10.1371/journal.pone.0152579 (PMC4809599; doi:10.1371/journal.pone.0152579)

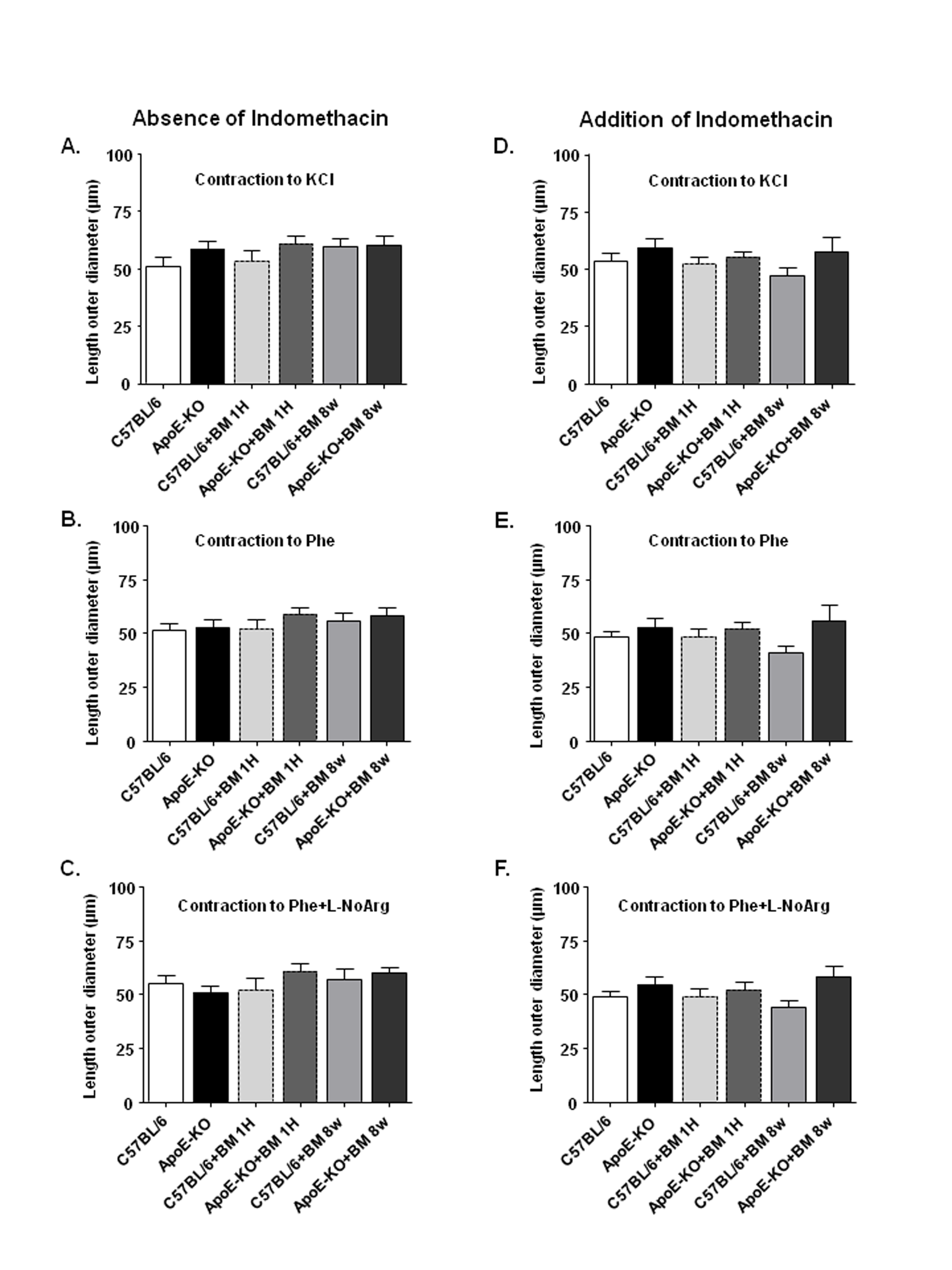

Supplement: S1 Fig — (A,D) Contractile response to KCl (50mM) or (B-F) phenylephrine (Phe) (10μM) in presence or absence of indomethacin (10μM) was measured in resistance mesenteric arteries from 15-week-old mice treated or not with BM-573 either per os during 8 weeks (10mg/L) or ex-vivo for 1H (3μM). No significant variations have been recorded in the contraction levels. Results are expressed as mean ± SEM (N = 5–8 animals in each group). (TIF) [file pone.0152579.s001.tif]

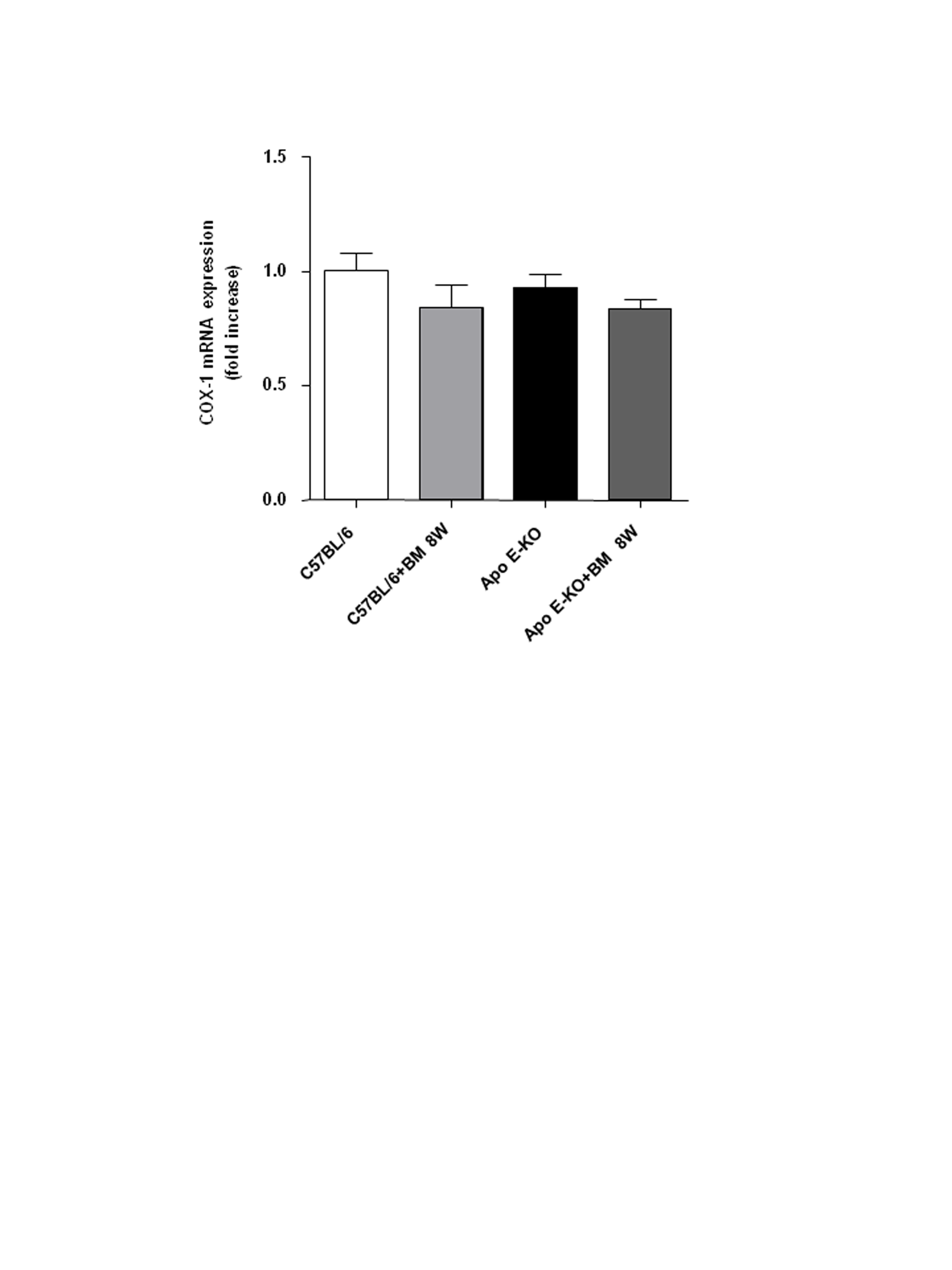

Supplement: S2 Fig — Quantitative real-time PCR-based analysis of COX-1 expression in aortic homogenates from C57BL/6J and ApoE-KO mice treated or not with BM-573. Results are expressed as mean ± SEM (N = 3–5 animals in each group). (TIF) [file pone.0152579.s002.tif]

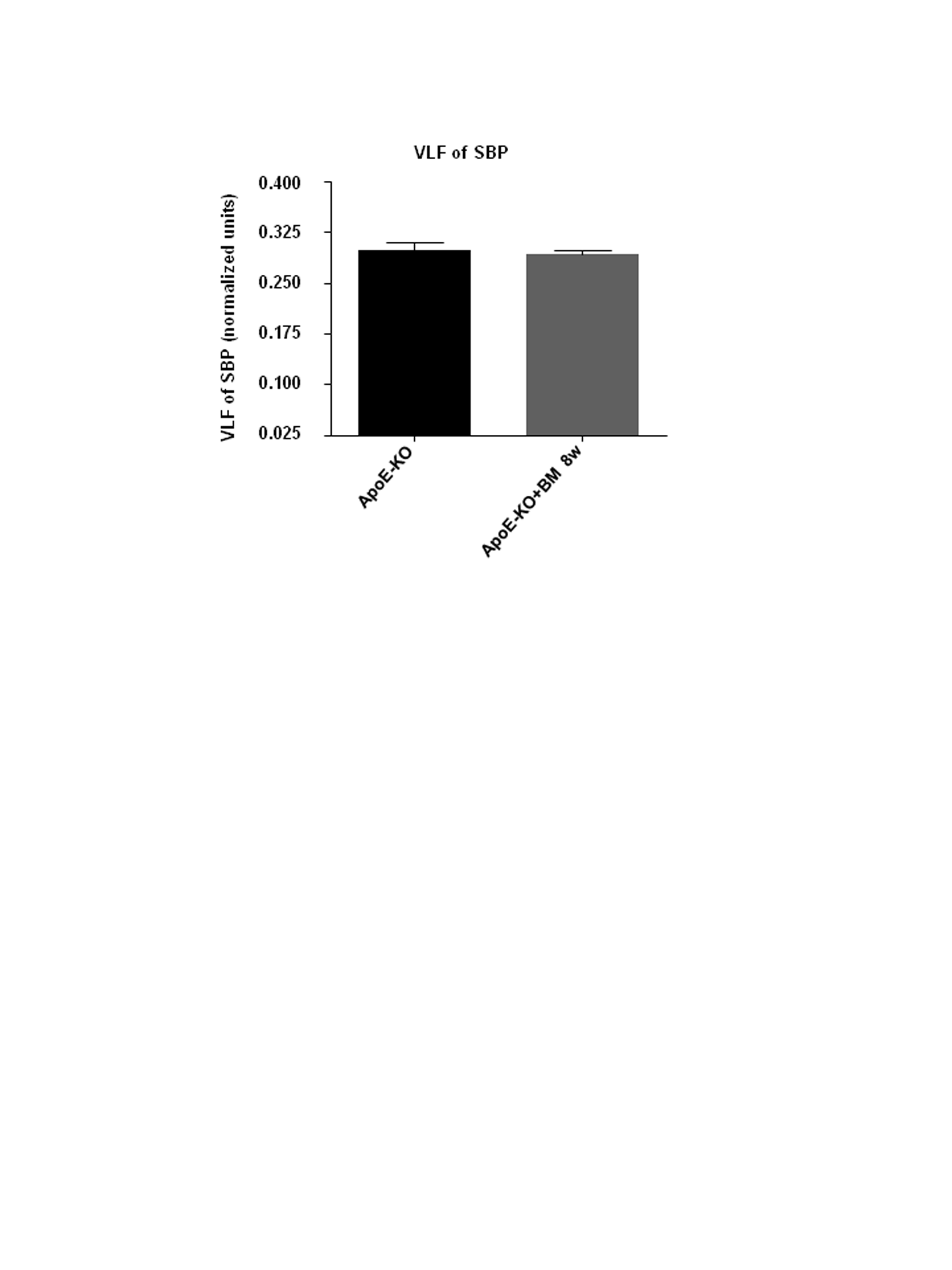

Supplement: S3 Fig — Effects of long-term BM-573 administration on blood pressure variability, specifically the very low frequency VLF band, measured by telemetry in ApoE-KO mice. Values are expressed as mean ± SEM (N = 3–5 animals in each group). (TIF) [file pone.0152579.s003.tif]
