# Supplementary material for: Effects of BM-573 on Endothelial Dependent Relaxation and Increased Blood Pressure at Early Stages of Atherosclerosis
Source: PLoS One. 2016 Mar 28;11(3):e0152579. doi: 10.1371/journal.pone.0152579 (PMC4809599; doi:10.1371/journal.pone.0152579)
Supplement: S1 File — This complete supplemental information file contains: (i) Drugs and buffers information; and (ii) methodological details relating to measurements of superoxide anion production by EPR, western blotting, quantitative real-time PCR and blood pressure and heart rate monitoring. (DOC) [file pone.0152579.s004.doc]

**Effects of BM-573 on Endothelial Dependent Relaxation and Increased Blood Pressure at Early Stages of Atherosclerosis.**

Short Title: Effects of BM-573 at Early Stages of Atherosclerosis.

Miguel Romero^1^, Elvira Leon-Gomez^1^, Géraldine Rath^1^, Irina Lobysheva^1^, Jean-Michel Dogné^2^, Olivier Feron^1^ and Chantal Dessy^1^.

**Supporting Information**

**Materials and Methods**

**Drugs and buffers**

BM-573 (N-tert-butyl-N’-[2-(4’-methylphenylamino)-5-nitrobenzenesulfonyl] urea) was synthesized in the Laboratory of Medicinal Chemistry of the University of Liège (Belgium). Others drugs and reagents were from Sigma, except DAPI from Calbiochem. BM-573 and indomethacin were initially dissolved in ethanol and sodium carbonate 2%, respectively. All other drugs were resuspended in distilled water.

PSS composition (in mM) 128.3 NaCl, 4.5 KCl, 1.35 CaCl2, 1.0 MgSO4, 0.35 KH2PO4, 11.0 glucose, and 20.23 NaHCO3, equilibrated at pH7.4 with a gas mixture of 95% O2 and 5% CO2.

**Measurements of superoxide anion production by EPR**

ROS formation was assayed by EPR using spin probe (1-Hydroxy-3-methoxycarbonyl-2,2,5,5-tetramethylpyrrolidine, CMH, ENZO Life Sciences Inc.) as offered previously with following modification: aortic rings (size about 1-1.5mm of length) were dissected from the same part of thoracic artery (close to abdominal artery), were preincubated on ice in KREBS-DTPA-Hepes buffer (0.1mM DTPA, 20mM HEPES, pH 7.5) with or without SOD (100U/mL), and inserted into the capillary immediately after addition of spin probe (CMH, 1mM CMH). Formation of CM. EPR signal was recorded during 10-15 minutes every 2 minutes at 37°C in capillary interposed into cavity of EPR spectrometer (MiniScop MS200, Magnetech equipped with bio-temperature controller). Following instrumental settings were used: modulation frequency, 100KHz; microwave frequency, 9.35GHz; microwave power, 20mW; modulation amplitude, 0.1mT. The amplitude of the second hf component of the triplet CM. EPR signal (AN = 16.1G) was used for quantitation. Level of aortic basal superoxide anion was calculated after subtraction of the rate of signal formation in presence of aortic ring preincubated with SOD from the rate of ring of nearby dissection, incubated without SOD. Rate was calculated by linearization of kinetic curve and normalized by length of aortic ring. All corresponding basal signals in buffer without aortic ring and with or without SOD were subtracted correspondingly to take into account only radical formation from aortic ring.

**Western blotting**

Isolated aorta were homogenized in lysis buffer (100mM Tris–HCl, pH 7.4, 50mM HEPES, 10mM EDTA, 100mM Na_4_P_2_O_7_, 100mM NaF, 10mM Na_3_VO_4_, 1% SDS). Western blots were performed with 30µg of protein per lane. Sodiumdodecyl sulfate-polyacrylamide (8%) electrophoresis was performed in a mini-gel system (Bio-Rad Laboratories). Phosphorylated protein kinase B (Akt) (Ser-473), phosphorylated eNOS (Ser-1177), Akt, eNOS and COX-2, were detected by incubation with the respective primary antibodies. Rabbit anti-p-eNOS-ser-1177 and rabbit anti-p-Akt-ser-473 were from Cell Signalling Technology, MA, USA; mouse anti-eNOS and mouse anti-Akt were from BD Biosciences, MA, USA and goat anti-COX-2 was from Santa Cruz Biotechnology, Santa Cruz, USA. All were used at 1/1000 dilution and incubated overnight at 4°C. The membranes were then incubated with secondary peroxidase conjugated goat anti-rabbit, goat anti-mouse or anti-goat antibodies (1:3000, Santa Cruz Biotechnology, Santa Cruz, USA), respectively. Antibody binding was detected by a chemiluminescent system (A mersham Pharmacia Biotech, Amersham, UK). Films were scanned and densitometric analysis was performed on the scanned images using Scion Image-Release Beta 4.02 software ([http://www.scioncorp.com](http://www.scioncorp.com/)).

**Quantitative real-time PCR**

The following specific primers were designed according to GenBank sequence and were as follows: NOX-1 forward: TGT GTC GAA ATC TGC TGT CC, and reverse: CAG AAG CGA GAG ATC CAT CC; and NOX-2 forward: GTT CCA GTG CGT GTT GCT C, and reverse: TGC AGT GCT ATC ATC CAA GC; and NOX-4 forward: AAC CTC AAC TGC AGC CTC AT, and reverse: CCT GCT AGG GAC CTT CTG TG; COX-1 forward: CAT CGC CAT GGA ATT TAA CC, and reverse: TCC TTG ATG ACA TCC ACA GC; and COX-2 forward: AGC AGA TGA CTG CCC AAC TC, and reverse: GGG TCA GGG ATG AAC TCT CTC. RPL13 (ribosomal protein L13) was used for the housekeeping gene, and specific primers were: RPL13 forward: CCT GCT GCT CTC AAG GTT GTT, and reverse: TGG TTG TCA CTG CCT GGT ACT T.

Ct (number of cycles needed to generate a fluorescent signal above a predefined threshold) was determined for each sample and the relative mRNA expression, expressed as fold variation, was calculated using the 2−ΔΔCt formula after normalization to housekeeping genes (ΔCt) and determination of the difference in Ct (ΔΔCt) between the various conditions tested.

**Blood pressure and Heart rate monitoring**

6-weeks-old male mice (≥20g) were kept under anaesthesia (Ketamine/Xylasine, 84 and 5mg/kg IP respectively) on a heating pad throughout implantation of the BP telemeter (model TA11PA- C10, Sciences International, St. Paul, MN). The left common carotid artery was isolated and the tip of the catheter was retrogradely inserted into the aorta until the aortic arch. The catheter was connected to the body of the implant, placed in a subcutaneous pouch in the right flank. Mice were individually housed and given analgesia (0.1mg/kg SQ BID) during 5 days before being placed on top of the telemetric receivers in a light-dark cycled recording room. When mice were 7-weeks-old, they were divided into two groups (n≥6) and were randomized to receive drinking water or BM-573 (10mg/L) in their drinking water for 8 weeks.

BP signals (and HR, derived from pressure waves) from the aortic arch were measured in conscious, unrestrained animals with surgically implanted, miniaturized telemetry devices. For each mouse, recording were done at 7 (basal level), 10, 12 and 15 (end of treatment) weeks.

The total spectral area was examined in each mouse. The area under the curve was calculated for frequencies below 0.05, 0.05-0.4Hz and above 0.4 Hz up to 5 Hz. Values were then examined for statistical changes between wild type and knock-out mice.
